# Dimethyl fumarate inhibits osteoclasts *via* attenuation of reactive oxygen species signalling by augmented antioxidation

**DOI:** 10.1111/jcmm.13367

**Published:** 2017-10-24

**Authors:** Yuuki Yamaguchi, Hiroyuki Kanzaki, Yuta Katsumata, Kanako Itohiya, Sari Fukaya, Yutaka Miyamoto, Tsuyoshi Narimiya, Satoshi Wada, Yoshiki Nakamura

**Affiliations:** ^1^ Department of Orthodontics School of Dental Medicine Tsurumi University Yokohama Japan

**Keywords:** osteoclast, Nrf2, dimethyl fumarate, receptor activator of nuclear factor‐kB ligand, oxidative stress, reactive oxygen species, antioxidant response, HO‐1

## Abstract

Bone destructive diseases are common worldwide and are caused by dysregulation of osteoclast formation and activation. During osteoclastogenesis, reactive oxygen species (ROS) play a role in the intracellular signalling triggered by receptor activator of nuclear factor‐κB ligand (RANKL) stimulation. Previously, we demonstrated that induction of antioxidant enzymes by Nrf2 activation using *Nrf2*‐gene transfer, an ETGE‐peptide or polyphenols, successfully ameliorated RANKL‐dependent osteoclastogenesis. Dimethyl fumarate (DMF) has been shown to activate Nrf2 signalling and has been lately used in clinical trials for neurodegenerative diseases. In this study, we hypothesized that Nrf2 activation by DMF would inhibit osteoclastogenesis and bone destruction *via* attenuation of intracellular ROS signalling through antioxidant mechanisms. RAW 264.7 cells were used as osteoclast progenitor cells. We found that DMF induced Nrf2 translocation to the nucleus, augmented Nrf2 promoter‐luciferase reporter activity and increased antioxidant enzyme expression. Using flow cytometry, we found that DMF attenuated RANKL‐mediated intracellular ROS generation, which resulted in the inhibition of RANKL‐mediated osteoclastogenesis. Local DMF injection into the calvaria of male BALB/c mice resulted in attenuated bone destruction in lipopolysaccharide‐treated mice. In conclusion, we demonstrated in a preclinical setting that DMF inhibited RANKL‐mediated osteoclastogenesis and bone destruction *via* induction of Nrf2‐mediated transcription of antioxidant genes and consequent decrease in intracellular ROS levels. Our results suggest that DMF may be a promising inhibitor of bone destruction in diseases like periodontitis, rheumatoid arthritis and osteoporosis.

## Introduction

Bone destructive diseases such as periodontitis, rheumatoid arthritis and osteoporosis are very common worldwide and are caused partly by dysregulation of osteoclast formation and activation 1. Osteoclasts are multi‐nucleated cells, differentiated from macrophages/monocytes, that resorb bone tissue 2, 3 under the stimulation of RANKL 4.

ROS mediate various signalling networks following RANKL stimulation 5 and may also exert cytotoxic effects, such as peroxidation of lipids and phospholipids 6 and oxidative damage to proteins and DNA 7, leading to tissue destruction. Cells possess several protective mechanisms against oxidative stress 8. One of the major cellular antioxidant responses is the induction of antioxidant enzymes *via* transcriptional control of nuclear factor E2‐related factor 2 (Nrf2) 9.

Previously, we reported that Nrf2 was transiently down‐regulated during osteoclastogenesis 10, 11. In these studies, we also found that overexpression of *Nrf2* inhibited osteoclastogenesis, and knockdown of *Nrf2* induced osteoclastogenesis. This is consistent with the results obtained by other groups that reported Nrf2 deficiency as inducer of osteoclastogenesis 12, 13. Furthermore, activation of Nrf2 by an ETGE‐peptide 14, polyphenols 15 or sodium hydrosulphide 16 was shown to inhibit osteoclastogenesis. Taken together, these results indicate that Nrf2 might be a key regulatory molecule of osteoclastogenesis.

As Nrf2 is the main regulator of antioxidant response and exerts also various other cytoprotective effect 8, chemicals that have the potential to activate Nrf2 have been extensively investigated 17, 18, 19. Fumarates, such as DMF, were shown to activate the Nrf2‐mediated signalling pathway 20, resulting in the activation of antioxidant enzymes such as haem oxygenase‐1 (HO‐1) 21, NAD(P)H Quinone Dehydrogenase 1 (NQO1) 22 and γ‐glutamylcysteine synthetase (GCS) 23. The ability of DMF to activate the antioxidant response leads to the idea to use DMF as the therapeutic drug against oxidative stress‐mediated diseases including periodontitis 24. While the antioxidant activity of DMF has been extensively reported, it remains unknown whether DMF exerts an anti‐osteoclastogenic effect.

In this study, we hypothesized that DMF induces the expression of antioxidant enzymes in osteoclast precursors, inhibits intracellular ROS levels and redox signalling networks and thereby attenuates osteoclastogenesis. To test this hypothesis, we performed *in vitro* experiments using RAW 264.7 cells as osteoclast precursor cells, as well as *in vivo* experiments using a mouse calvarial bone destruction model.

## Materials and methods

### Chemicals

Recombinant RANKL and DMF were purchased from Wako Pure Chemical (Osaka, Japan), and dissolved in ethanol. Vehicle control for DMF experiments was 0.1% ethanol. Purified lipopolysaccharide (LPS) from *Escherichia coli* O111:B4 (Sigma‐Aldrich, St. Louis, MO, USA) was dissolved in PBS at a concentration of 1 mg/ml.

### Cells

The mouse monocytic cell line, RAW 264.7, was obtained from the Riken Bioresource Center (Tsukuba, Japan).

### Cell culture

RAW 264.7 cells were cultured in alpha‐modified Eagle's medium (Wako Pure Chemical, Osaka, Japan) that contained 10% foetal bovine serum (Thermo Fisher Scientific, Waltham, MA) and supplemented with penicillin (100 U/ml) and streptomycin (100 μg/ml). All cells were cultured at 37°C in a 5% CO_2_ incubator.

### Cell viability assay

DMF‐mediated cytotoxicity was assessed using a cell counting kit‐8 (CCK‐8; Dojindo, Tokyo, Japan). In brief, RAW 264.7 cells were plated in 24‐well plates and cultured with DMF (0, 1, 10, and 100 μM) for 1 day. The kit reagent was added to cultures, and optical density at 450 nm (OD450) was measured using the Synergy HTX Multi‐Mode plate Reader (BioTek Japan, Tokyo, Japan) after 1‐hr incubation.

### Preparation of nuclear and cytoplasmic protein lysate

The method used for preparation of nuclear and cytoplasmic protein lysate had been previously described by us 10. Briefly, nuclear protein lysate was prepared from RAW 264.7 cells using the DUALXtract cytoplasmic and nuclear protein extraction kit (Dualsystems Biotech AG, Schlieren, Switzerland) according to the manufacturer's instructions. Cultured cells were washed with PBS and treated with cell lysis buffer. After centrifugation, the nuclear pellet was washed twice and lysed with nuclear lysis reagent. After centrifugation, the cleared supernatant was used as nuclear protein extract. For nuclear NRF2 Western blot analysis, nuclear protein samples were extracted after 6 hrs of DMF treatment. For Western blot analysis of cytoplasmic protein, samples were extracted after 1 day of DMF treatment. Briefly, the cytoplasmic protein samples were prepared using lysis buffer (5 mM EDTA, 10% glycerol, 1% Triton X‐100, 0.1% SDS, 1% NP‐40 in PBS) containing proteinase inhibitor cocktail (Wako Pure Chemical, Osaka, Japan). Protein concentration in each of the lysates was measured with the Quick Start Protein Assay Kit (Bio‐Rad Laboratories; for nuclear lysates) or with Pierce BCA Protein Assay Kit (Thermo Fisher Scientific, Waltham, MA; for cytoplasmic lysates), and adjusted to be the same for each lysate. After mixing with sample buffer‐containing 2‐mercaptoethanol (2‐ME), samples were heat‐denatured and subjected to electrophoresis and Western blotting.

### Western blot analysis

Prepared cellular lysates, which contained equal amounts of protein, were subjected to electrophoresis on TGX Precast gels (Bio‐Rad), proteins were transferred to a PVDF membrane, which was blocked with PVDF Blocking Reagent (Toyobo Co. Ltd, Osaka, Japan), then incubated with the primary antibody (Ab). After thorough washing with 0.5% Tween‐20 in PBS (PBS‐T), the membrane was incubated with a horseradish peroxidase‐conjugated secondary Ab. Chemiluminescence was produced using Luminata‐Forte (EMD Millipore, Billerica, MA) and detected with LumiCube (Liponics, Tokyo, Japan). The primary antibodies for these experiments were anti‐Nrf2 (1/1000 dilution; Santa Cruz Biotechnology Inc.), anti‐histone H3 (1/4000; Cell Signaling Technology Japan, Tokyo, Japan), anti HO‐1 (1/5000; StressMarq Biosciences Inc., Victoria, BC, Canada), anti‐NQO1 (1/5000; Abcam plc, Cambridge, MA, USA) and anti‐GCS (1/5000; Thermo Fisher Scientific Inc.).

### DNA transfection and luciferase activity assay

RAW 264.7 cells were transfected with a Nrf2‐responsive luciferase construct (Cignal Antioxidant Response Reporter; Qiagen, Germantown, MD) using X‐tremeGENE HP DNA transfection reagent (Roche, Tokyo Japan) in a reverse transfection format. Briefly, the Nrf2‐responsive luciferase construct (0.1 μg) and the transfection reagent (1 μl) were mixed in serum‐free medium (120 μl) in 24‐well plates then incubated at room temperature for 0.5 hrs. Then, a RAW 264.7 cell suspension (2 × 10^5 ^cells/well, 500 μl/well) was added to the plate, and the cells were incubated overnight. The next day, cells were cultured with 0.1% ethanol or 10 μM of DMF for 24 hrs, and cell lysates were prepared using lysis buffer‐containing long half‐life luciferase substrate (Pikka‐gene LT7.5; Toyo B‐Net, Tokyo, Japan). Firefly luciferase activities were measured with a Synergy HTX Multi‐Mode plate Reader (BioTek Japan, Tokyo, Japan).

### Real‐time RT‐PCR analysis

RNA was extracted from RAW 264.7 cells using the GenElute mammalian total RNA Miniprep kit (Sigma‐Aldrich) with an on‐column genomic DNA digestion. For antioxidant gene expression analysis, RNA was extracted 1 day after the treatment of cells with 10 μM of DMF treatment, and the gene expression of *Nqo1*,* Ho‐1* and *Gcs* was analysed.

For osteoclast marker gene expression analysis, RNA was extracted at 4 days after 100 ng/ml of RANKL stimulation of RAW 264.7 cells, and the gene expressions of *Atp6v0d2*,* Cathepsin K* (*Ctsk*), *matrix metalloproteinase 9* (*Mmp9*) and *tartrate‐resistant acid phosphatase* (*Trap*) were analysed.

Isolated RNA (500 ng each) was reverse transcribed with iScript cDNA‐Supermix (Bio‐Rad, Hercules, CA, USA). Real‐time RT‐PCR was performed with SsoFast EvaGreen‐Supermix (Bio‐Rad). PCR primers used in the experiments were from PrimerBank (Boston, MA, USA) and have been described previously 10. Fold changes of genes of interest were calculated using the Δ‐Δ Ct method with *ribosomal protein S18* (*RPS18*) was used as a reference gene.

### Intracellular ROS detection

RAW 264.7 cells were pretreated with or without 10 μM of DMF for 1 hr and stimulated with recombinant RANKL (100 ng/ml) for another 6 hrs, then washed and harvested. The cell suspension was thereafter incubated with a fluorescent superoxide probe (BES‐So‐AM, 5 μM final; Wako Pure Chemical) in PBS containing 2% FBS on ice for 30 min. After washing three times with PBS containing 2% FBS, intracellular ROS was detected using an AccuriC6 flow cytometer (BD Biosciences, San Jose, CA), and data were processed using an analysis software, FlowJo (FlowJo, LLC, Ashland, OR, USA). The viable cellular fraction of monocyte/macrophage was gated on a forward scatter/side scatter plot, and intracellular ROS levels were monitored in the FL‐1 channel.

### Monocyte/macrophage marker detection by flow cytometry

RAW 264.7 cells were treated with DMF (10 μM) for 1 day and collected. PerCP/Cy5.5‐conjugated anti CD11b antibody (1/100 dilution; BioLegend, San Diego, CA) was added into the cell suspension and incubated for 30 min on ice. The cells were then washed three times, and the expression of CD11b was detected using an AccuriC6 flow cytometer (BD Biosciences). Data were processed using an analysis software, FlowJo.

### Osteoclastogenesis assay

RAW 264.7 cells were plated on 24‐well plates (5 × 10^3 ^cells/well) in the presence or absence of recombinant RANKL (100 ng/ml) in the presence and absence of DMF (0, 0.1, 1, and 10 μM). After 4 days of culture, cells were stained for TRAP using an acid phosphatase kit (Sigma‐Aldrich). Dark red multi‐nucleated cells (over three nuclei) were counted as TRAP‐positive, multi‐nucleated cells.

### *In vivo* bone destruction model

All animal study protocols were reviewed and approved by the Institutional Animal Care and Use Committee of Tsurumi University (No. 28A030). Animal experiments were performed in compliance with the Regulations for Animal Experiments and Related Activities at Tsurumi University. The calvarial bone destruction mouse model, induced by repeated LPS injections, has been described previously 14. Twenty 7‐week‐old male BALB/c mice (Clea Japan, Tokyo, Japan) were used. Mice were randomly assigned to four groups (*n* = 5 each): a PBS‐injected group (control group; 10 μl PBS + 2 μl 0.1% ethanol), a DMF‐injected group (DMF group; 10 μl PBS + 2 μl of DMF 10 mM), an LPS‐induced bone resorption group (LPS group; 10 μl of 1 mg/ml LPS + 2 μl 0.1% ethanol) and an LPS‐induced bone resorption and DMF‐injected group (LPS + DMF group; 10 μl of 1 mg/ml LPS + 2 μl of DMF 10 mM). Injections were performed under anaesthesia with a 30‐gauge needle at a point on the midline of the skull located between the eyes on days 1, 3, 5, 7 and 9. On day 11, mice were killed by cervical dislocation and cranial tissue samples were fixed overnight with 4% paraformaldehyde in PBS.

### Micro‐computed tomography analysis for bone destruction

Fixed cranial tissue samples were subsequently scanned with an X‐ray micro‐computed tomography (microCT) system (inspeXio SMX‐225CT; Shimadzu Corp., Kyoto, Japan). After reconstitution, the DICOM files were rendered into three‐dimensional images using the OsiriX 64 bit (Newton Graphics, Sapporo, Japan). Percentage of resorbed area, calculated from the ratio of the number of pixels in the resorbed area in the cranial bone to the number of pixels in the total analysed image of the cranial bone, was calculated with the ImageJ software (National Institutes of Health, Bethesda, MD, USA). The region of interest was set between the fronto‐parietal (coronal) suture and parieto‐occipital (lambdoidal) suture.

### Statistical analysis

All data are presented as means ± S.D. Multiple comparisons were performed using the Tukey's test. A value of *P *<* *0.05 was considered to be statistically significant.

## Results

### Assessment of DMF‐mediated cytotoxicity

**Figure 1:**
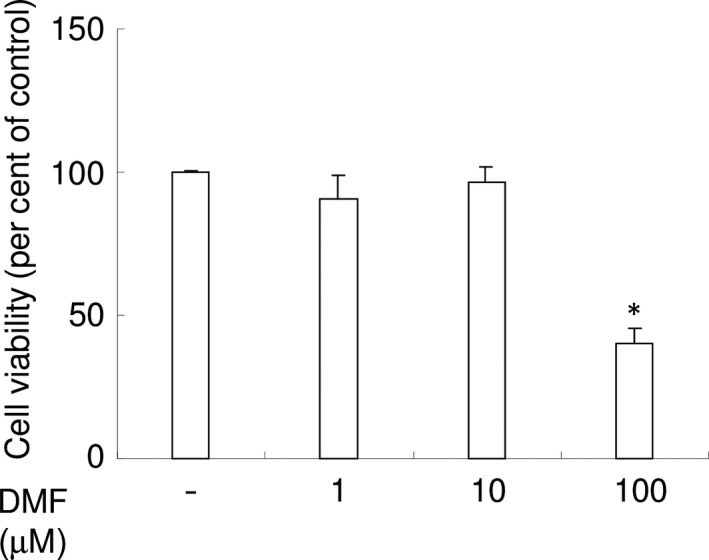
DMF exhibits no cytotoxicity against RAW 264.7 cells at concentrations <10 μM. Cytotoxicity of several concentrations of DMF was examined using CCK‐8. Per cent of control were shown. *P <* *0.05 *versus* control.

We first examined whether DMF exhibits cytotoxicity against RAW 264.7 cells. The highest tested concentration of DMF (100 μM) exhibited cytotoxicity, with approximately 50% cell viability compared with the vehicle control (Fig. 1). At 10 μM DMF, there was no statistically significant difference in cell viability, and hence, we used this DMF concentration for subsequent experiments.

### DMF induces nuclear Nrf2 translocation and augments Nrf2 promoter‐luciferase reporter activity

**Figure 2:**
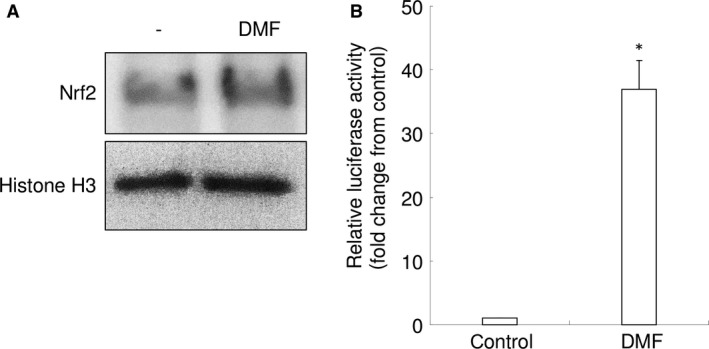
DMF (10 μM) induces nuclear Nrf2 translocation and Nrf2‐responsive luciferase activity in RAW 264.7 cells. (**A**) Western blot analysis of Nrf2 and histone H3 using nuclear protein lysates. Representative images are shown. (**B**) Relative activity of the Nrf2‐responsive luciferase in RAW 264.7 cells. *P <* *0.05 *versus* control.

We examined whether DMF induces nuclear Nrf2 translocation in RAW 264.7 cells. Western blotting using nuclear extracts of RAW 264.7 cells clearly demonstrated that stimulation of RAW 264.7 cells with 10 μM of DMF led to Nrf2 translocation to the nucleus (Fig. 2A).

Using a Nrf2 promoter‐luciferase reporter assay, we found that 10 μM of DMF induced antioxidant response element (ARE)‐dependent transcription of the reporter gene, indicating increased transcriptional activity of Nrf2 (Fig. 2B). These results suggest that DMF triggers by itself Nrf2‐mediated transcription of antioxidant molecules in RAW 264.7 cells.

### DMF induces the expression of antioxidant enzymes

**Figure 3:**
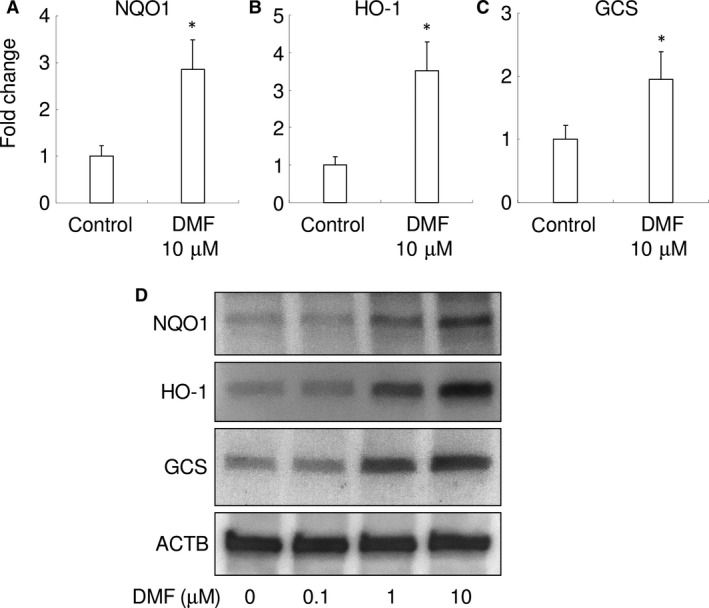
DMF increases expression of antioxidant enzymes in RAW 264.7 cells. Gene expression for *NQO1* (**A**), *HO‐1* (**B**) and *GCS* (**C**) are shown. *P <* *0.05 *versus* control. (**D**) Representative images of Western blot analysis for *NQO1*,*HO‐1*,*GCS* and *ACTB* are shown.

To further examine the effect of DMF on the antioxidant response, we examined the expression of NRF2 gene targets antioxidant enzymes in RAW 264.7 cells, such as *NQO1*,* HO‐1* and *GCS*. The mRNA of *NQO1*,* HO‐1* and *GCS* were up‐regulated by 10 μM of DMF in RAW 264.7 cells (Fig. 3A–C). Protein expression of these antioxidant enzymes was up‐regulated by DMF, in a dose‐dependent manner (Fig. 3D). These results indicate that DMF substantially induces an antioxidant response in RAW 264.7 cells.

### DMF attenuates RANKL‐mediated intracellular ROS

**Figure 4:**
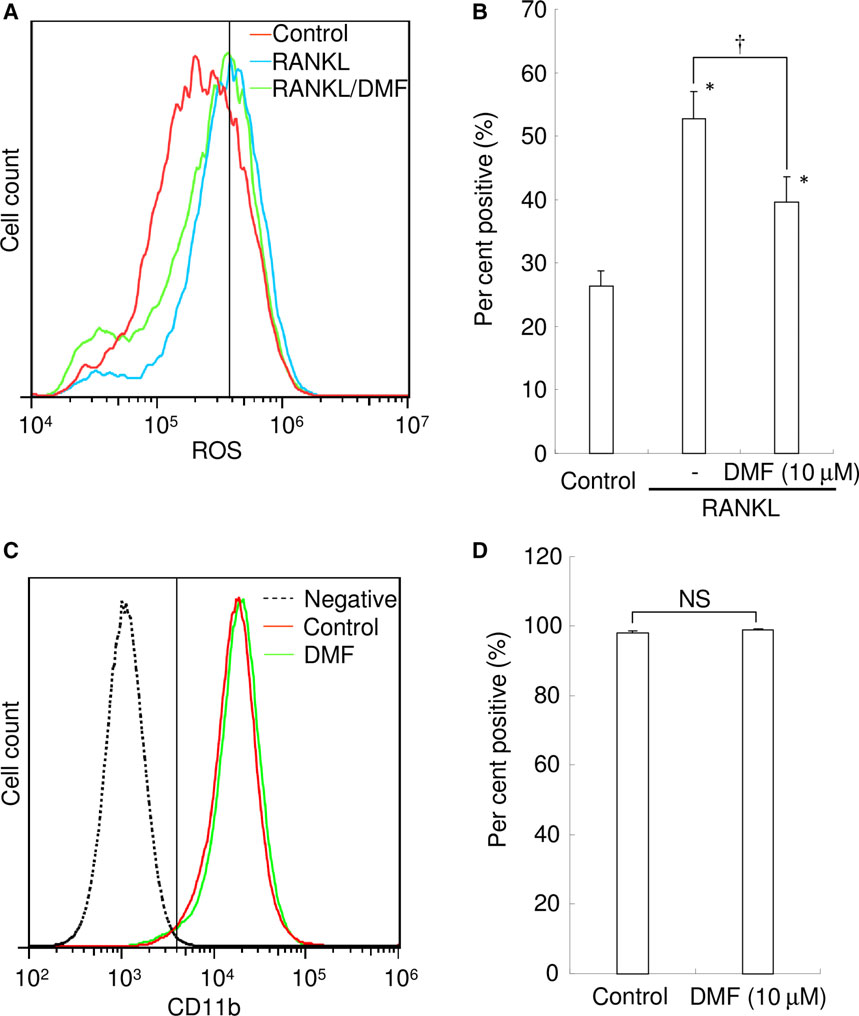
DMF attenuates RANKL‐mediated intracellular ROS levels but has no impact on monocyte‐specific markers in RAW 264.7 cells. (**A**) Intracellular ROS levels in control (red), RANKL‐treated (blue) and RANKL‐ and DMF‐treated RAW 264.7 cells (green) are shown. The vertical line indicates a conventional threshold for ROS‐negative and ‐positive populations. (**B**) Mean per cent of ROS‐positive RAW 264.7 cells. 10 μM of DMF was used. *P <* *0.05 *versus* control. ^†^ *P *<* *0.05 between groups. (**C**) CD11b expression in negative control (unstained; black dotted line), control (red) and DMF (10 μM)‐treated RAW 264.7 cells (green). The vertical line indicates the threshold for CD11b‐negative and ‐positive populations. (**D**) Mean per cent of CD11b‐positive RAW 264.7 cells. NS: not significant.

Next, we used flow cytometry to investigate whether DMF could interfere with RANKL‐triggered intracellular ROS production in RAW 264.7 cells (Fig. 4A and B). Treatment of RAW 264.7 cells with RANKL (100 ng/ml) increased intracellular production of superoxide, as detected using BES‐So‐AM. Treatment with 10 μM of DMF inhibited this RANKL‐mediated increase in intracellular ROS (Fig. 4A and B). Results suggest that DMF attenuates RANKL signalling, resulting in increased superoxide production.

### DMF does not affect CD11b expression

To determine if DMF affects monocyte/macrophage differentiation in RAW 264.7 cells, we analysed CD11b expression, a monocyte/macrophage marker, by flow cytometry. There was no difference between control and DMF (10 μM)‐treated cells (Fig. 4C and D), indicating that DMF had no impact on monocytic markers expressed by RAW 264.7 cells.

### DMF inhibits RANKL‐mediated osteoclastogenesis in a dose‐dependent manner

**Figure 5:**
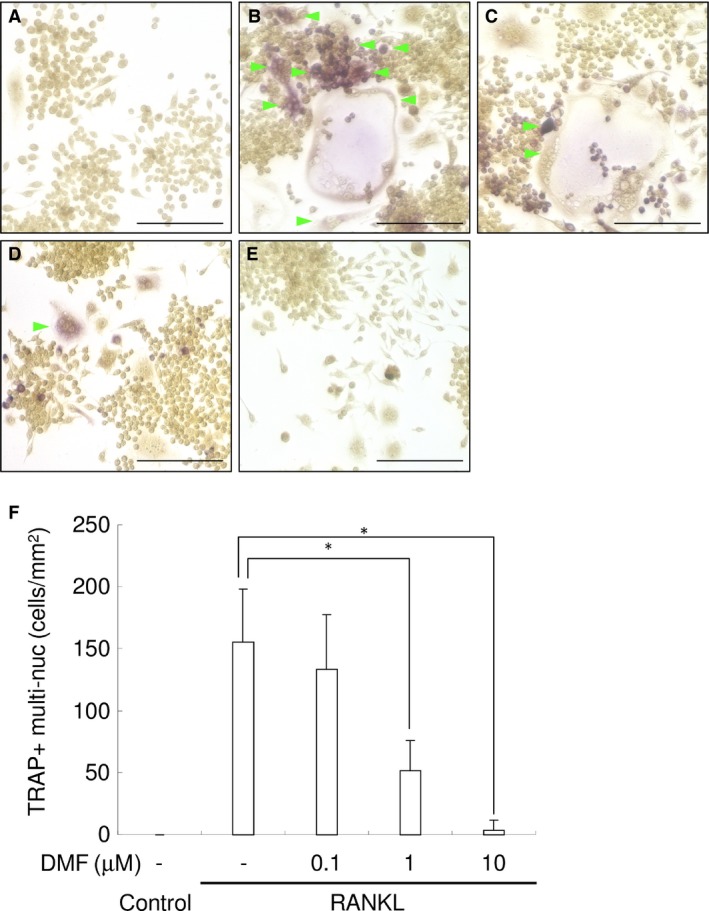
DMF inhibits RANKL‐mediated osteoclastogenesis in a dose‐dependent manner. Representative photographs of control (**A**), RANKL‐treated (**B**), RANKL+DMF (0.1 μM) (**C**), RANKL+DMF (1 μM) (**D**) and RANKL+DMF (10 μM) (**E**) are shown. Green arrowhead indicates TRAP‐positive multi‐nucleated cells. Scale bars: 100 μm. (**F**) Mean number of TRAP‐positive multi‐nucleated cells. *P <* *0.05 between samples.

We next examined whether DMF exhibited an inhibitory effect on RANKL‐mediated osteoclastogenesis. RANKL (100 ng/ml) stimulation of RAW 264.7 cells induced a high number of TRAP‐positive, multi‐nucleated cells, compared with the control, indicating increased osteoclastogenesis (Fig. 5A and B). DMF dose‐dependently inhibited RANKL‐mediated osteoclastogenesis, as demonstrated by a significant reduction in TRAP‐positive, multi‐nucleated cells (Fig. 5C–E). The highest tested concentration of DMF (10 μM) almost completely inhibited RANKL‐mediated osteoclastogenesis (Fig. 5F). These results indicate that DMF directly inhibits osteoclast differentiation in RAW 264.7 cells *in vitro*.

### DMF inhibits osteoclast function

**Figure 6:**
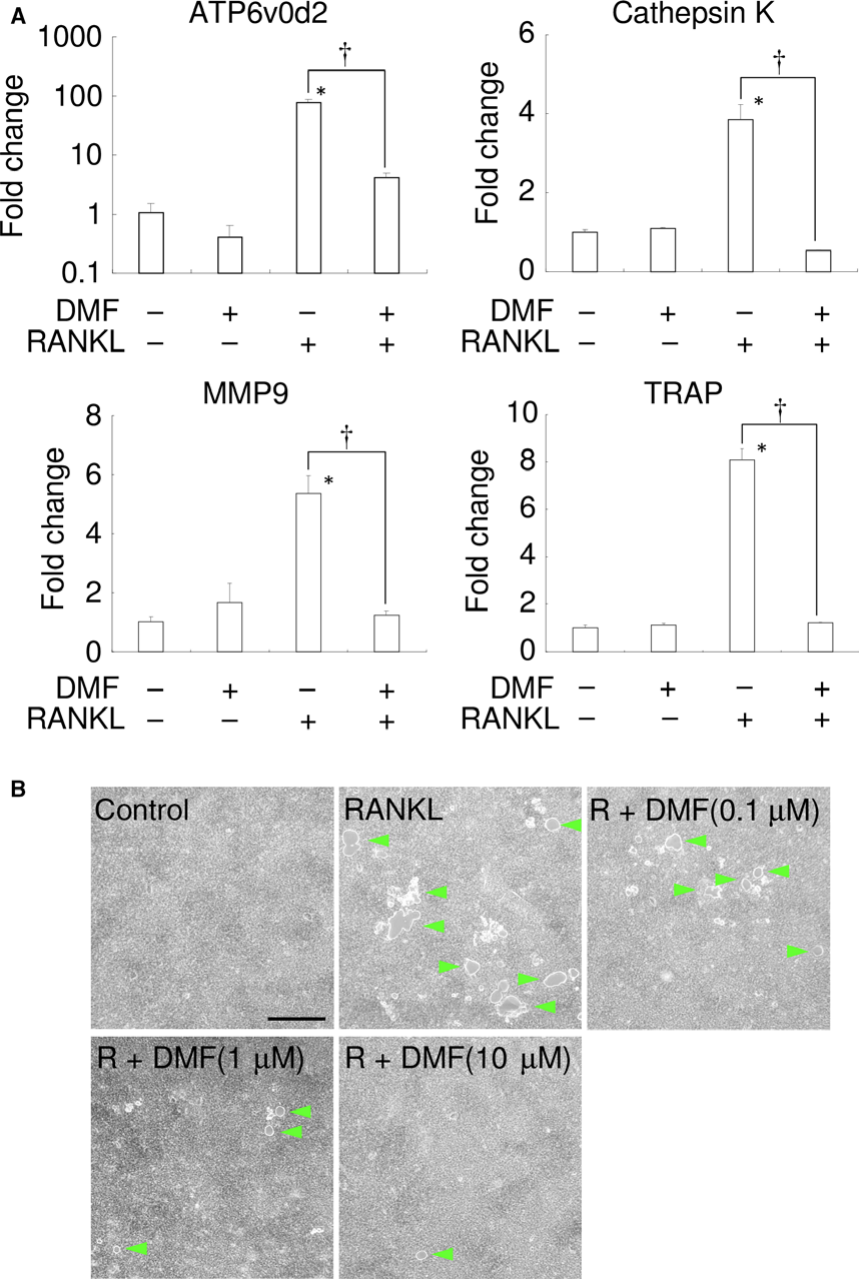
DMF inhibits the function of osteoclasts. (**A**) The results of real‐time RT‐PCR analysis of the RAW 264.7 cells genes for *ATP6V0D2*,*CTSK*,*MMP9* and *TRAP* are shown following treatment with 100 ng/ml recombinant RANKL and 10 μM of DMF. *P <* *0.05 *versus* control. ^†^ *P *<* *0.05 between samples. (**B**) Resorption assay. Representative photographs of the control, RANKL‐treated, RANKL+DMF (0.1 μM), RANKL+DMF (1 μM) and RANKL+DMF (10 μM) are shown. Green arrowhead indicates resorbed area. 100 ng/ml recombinant RANKL was used. Scale bars: 500 μm.

To further examine the *in vitro* effects of DMF on osteoclasts at molecular level, the gene expression of several osteoclast differentiation markers in RAW 264.7 cells was examined by real‐time RT‐PCR. RANKL induced the expression of *ATP6V0D2*,* CTSK*,* MMP9* and *TRAP* (Fig. 6A). Treatment with 10 μM of DMF almost completely inhibited the RANKL‐mediated induction of these osteoclastic differentiation markers with no significant difference between control cells and RANKL/DMF‐treated cells in all the tested genes.

Next, resorption activity was examined using the bone resorption assay plate (Fig. 6B). RANKL stimulation of RAW 264.7 cells induced numerous resorption areas on the substrate, and DMF dose‐dependently reduced the resorption areas. These results suggest that DMF (10 μM) inhibited not only osteoclastogenesis, but also osteoclast activity.

### Local DMF injection ameliorates LPS‐induced bone destruction in mice

**Figure 7:**
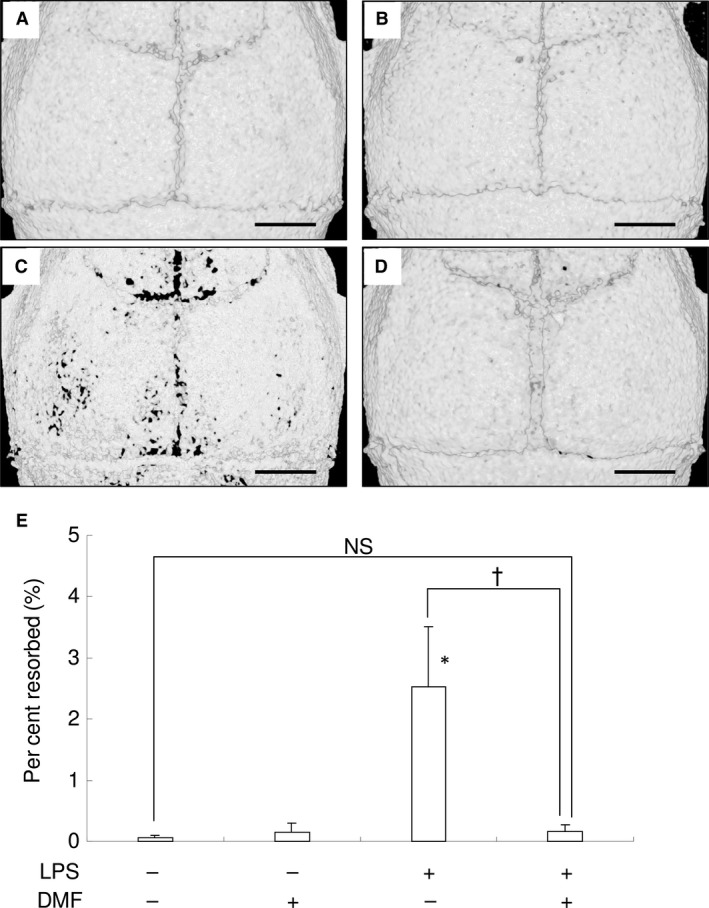
Local DMF injection ameliorates LPS‐induced bone destruction in mice. (**A–D**) Representative microCT image of control (**A**), DMF (**B**), LPS (**C**) and LPS+DMF‐treated mice (**D**). DMF (20 nM/site) were used. Scale bar: 1 mm. (**E**) Percentage of resorbed area in the cranial bone. NS: no significant difference between groups. *P <* *0.05 *versus* control. ^†^ *P *<* *0.05 between groups.

Finally, we examined whether local DMF injection can ameliorate LPS‐mediated bone destruction in mice calvaria. Repeated LPS injection (five times, every other days, total 50 μg of LPS) induced bone destruction in mice compared with the control group (Fig. 7A and C). Five local DMF injections ameliorated LPS‐mediated bone destruction as demonstrated by microCT imaging of resorbed areas in calvaria (Fig. 7C and D). We measured resorbed areas and found that DMF (20 nMol/site) almost completely inhibited LPS‐mediated bone destruction (Fig. 7E). These results indicate that DMF is a potential inhibitor against bone destruction in preclinical settings.

## Discussion

In this study, we showed for the first time that DMF, a Nrf2 activator, inhibited RANKL‐mediated osteoclastogenesis and bone destruction. DMF transcriptionally up‐regulated the expression of Nrf2‐mediated antioxidant enzymes and decreased RANKL‐mediated ROS generation in RAW 264.7 cells. RANKL‐mediated ROS plays an important role in the osteoclastogenesis and has been a candidate target for bone destructive diseases. The signalling cascade from RANK to ROS in the osteoclastogenesis consists of TRAF6, Rac1 and NOX 9. In addition, the downstream events of ROS consist of MAPK, PI3K and NF‐kB activation 9. Therefore, DMF decreases the ROS signalling by scavenging with augmented antioxidant enzymes in RAW 264.7 cells and consequently inhibits osteoclastogenesis. This was supported by the *in vivo* experiments that DMF exhibited the inhibitory effect on LPS‐mediated bone destruction. These results suggest that DMF is a potential inhibitor of bone destruction in diseases like periodontitis, rheumatoid arthritis and osteoporosis.

DMF is known to alkylate numerous proteins, including Kelch‐like ECH‐associated protein 1 (Keap1), a repressor of Nrf2, and thereby protect Nrf2 from Keap1‐mediated ubiquitination and degradation 25. Besides DMF, there are several other molecules known to disrupt the Keap1/Nrf2 interaction 19. Among them, Bardoxolone (CDDO) is a well‐known small molecule that activates Nrf2, but unlike DMF, its complicated pharmacological and toxicological profiles raise serious clinical issues 26. As DMF promotes cytoprotection against oxidative stress *via* the Nrf2 pathway 27, DMF was thought to have therapeutic potential against oxidative stress‐mediated diseases, such as multiple sclerosis 28. Tecfidera™ (BG‐12; Biogen, Research Triangle Park, NC, USA), an oral formulation of DMF, is prescribed to patients with multiple sclerosis, a multifocal inflammatory demyelinating disease of the central nervous system, and showed remarkable efficacy in lowering relapse rates of multiple sclerosis in clinical trials *via* antioxidant mechanisms 29. The phase 3 clinical trial of DMF for multiple sclerosis found a significant reduction in relapse rates and improved neuroradiologic outcome relative to placebo 30, signifying that DMF is safe and effective for clinical use as Nrf2 inducer.

Our results demonstrate that a one‐time exposure of DMF to macrophages *in vitro,* and *in vivo* injection of DMF every second day exhibited a significant inhibitory effect on osteoclasts, even though the half‐life of DMF is approximately 12 min 31. We assume that the reason why DMF exhibited such a significant inhibitory effect despite its short half‐life was due to the long half‐life of monomethyl fumarate (MMF), the most bioactive metabolite of DMF. The half‐life of MMF is about 36 hrs 31, and we presumed that MMF might exert prolonged anti‐osteoclastogenic activity. Indeed, MMF exhibited anti‐osteoclastogenic activity at similar molecular concentration of DMF *in vitro* (data not shown). DMF has been shown to exhibit cytoprotective effects *via* the Nrf2‐pathway on central nervous system cells 27, liver cells 22 and epithelial cells 23. In addition, an immuno‐modulatory effect of DMF on human peripheral blood lymphocytes has also been reported 21, 32. More recently, it was reported that Nrf2 opposes transcriptional up‐regulation of pro‐inflammatory cytokine genes 33. Excessive immune response plays a role in the bone destruction in periodontitis 24. The immuno‐modulatory effect of DMF would be helpful to remit the production of osteoclastogenic cytokines in periodontitis, but this is yet to be experimentally confirmed.

Regarding the effects of DMF on monocytes/macrophages as osteoclast precursors, DMF was shown to suppress the infiltration of monocytes/macrophages into tissue 34, induce an alteration of macrophage M1/M2 polarization 35 and attenuate CCL2‐induced monocyte chemotaxis 36. However, until the present study, there has been no report on the effects of DMF on osteoclastogenesis. Our analysis demonstrated that DMF had no impact on CD11b expression in RAW 264.7 cells, indicating that the inhibitory effect of DMF against osteoclastogenesis is solely due to interference in intracellular RANKL signalling, and is not involved in monocytic differentiation. We examined the effect of DMF on CD11b expression in RAW 264.7 cells, which is recognized as the pan‐macrophage marker 37. Therefore, the discrepancy between our results and the previous reports would be due to the difference in the observed marker. Further investigations are necessary to clarify the relationship between DMF‐mediated change of phenotype of macrophage and osteoclastogenesis.

As shown above, our results demonstrated that DMF can inhibit osteoclastogenesis. Other Nrf2 activators, such as the ETGE‐peptide 14, sulforaphane and polyphenols 15, were also shown to inhibit osteoclastogenesis through NRF2 activation. These results clearly indicate that the Nrf2 transcription factor is a potential pharmacological target for osteoclast inhibition. Nevertheless, bone metabolism is based on the balance between osteoclastic bone resorption and osteoblastic bone formation 38. The effects of Nrf2 activation on osteoblastic bone formation should be also considered.

The effects of Nrf2 activation on osteoblast differentiation are controversial. Hinoi *et al*. reported that Nrf2 inhibited osteoblast differentiation 39. Global *Nrf2* knockout in mice increased the mineral apposition rate 40. On the other hand, bone marrow stromal cells from *Nrf2* knockout mice failed bone acquisition 41. *Nrf2* knockout mice also had impaired bone metabolism and diminished load‐driven bone formation 42. As oxidative stress itself inhibits osteoblast differentiation and bone formation 39, 43, ROS scavenging would protect osteoblast from oxidative stress. Indeed, regulation of the intracellular ROS levels promoted osteoblastic differentiation in human periodontal ligament cells 44, 45 and osteoblasts 46, 47, 48. Taken together, further detailed studies are necessary to explore the effects of Nrf2 induction in osteoblasts by DMF.

In conclusion, we have demonstrated in a preclinical setting that DMF inhibits RANKL‐mediated osteoclastogenesis and bone destruction *via* attenuation of intracellular ROS signalling. Our results suggest that DMF is a potential inhibitor of pathologic bone destruction in diseases like periodontitis, rheumatoid arthritis and osteoporosis.

## Conflict of interest

The authors declare no conflict of interests.
